# Studies on Properties of Rice Straw/Polymer Nanocomposites Based on Polycaprolactone and Fe_3_O_4_ Nanoparticles and Evaluation of Antibacterial Activity

**DOI:** 10.3390/ijms151018466

**Published:** 2014-10-14

**Authors:** Roshanak Khandanlou, Mansor B. Ahmad, Kamyar Shameli, Elnaz Saki, Katayoon Kalantari

**Affiliations:** 1 Department of Chemistry, Faculty of Science, Universiti Putra Malaysia, 43400 UPM Serdang, Selangor, Malaysia; E-Mails: kamyarshameli@gmail.com (K.Sh.); Ka_Kalantary@yahoo.com (K.K.); 2 Department of Pathology, Faculty of medicine and Health Sciences, Universiti Putra Malaysia, 43400 UPM Serdang, Selangor, Malaysia; E-Mail: E.saki65.e@gmail.com

**Keywords:** polycaprolactone, rice straw/Fe_3_O_4_ nanocomposites, X-ray powder diffraction, scanning and transmission electron microscopy, antibacterial activity

## Abstract

Modified rice straw/Fe_3_O_4_/polycaprolactone nanocomposites (ORS/Fe_3_O_4_/PCL-NCs) have been prepared for the first time using a solution casting method. The RS/Fe_3_O_4_-NCs were modified with octadecylamine (ODA) as an organic modifier. The prepared NCs were characterized by using X-ray powder diffraction (XRD), Scanning electron microscopy (SEM), Transmission electron microscopy (TEM), Thermogravimetric analysis (TGA) and Fourier transform infrared spectroscopy (FT-IR). The XRD results showed that as the intensity of the peaks decreased with the increase of ORS/Fe_3_O_4_-NCs content in comparison with PCL peaks, the Fe_3_O_4_-NPs peaks increased from 1.0 to 60.0 wt. %. The TEM and SEM results showed a good dispersion of ORS/Fe_3_O_4_-NCs in the PCL matrix and the spherical shape of the NPs. The TGA analysis indicated thermal stability of ORS/Fe_3_O_4_-NCs increased after incorporation with PCL but the thermal stability of ORS/Fe_3_O_4_/PCL-NCs decreased with the increase of ORS/Fe_3_O_4_-NCs content. Tensile strength was improved with the addition of 5.0 wt. % of ORS/Fe_3_O_4_-NCs. The antibacterial activities of the ORS/Fe_3_O_4_/PCL-NC films were examined against Gram-negative bacteria (*Escherichia coli*) and Gram-positive bacteria (*Staphylococcus aureus*) by diffusion method using nutrient agar. The results indicated that ORS/Fe_3_O_4_/PCL-NC films possessed a strong antibacterial activity with the increase in the percentage of ORS/Fe_3_O_4_-NCs in the PCL.

## 1. Introduction

Currently, the applications of natural products as eco-friendly materials, in waste disposal have gained a great deal of attention from researchers, particularly, for the synthesis of composites. Polycaprolactone (PCL) as a biodegradable and biocompatible polyester with high potential of application is used in many areas such as agricultural usage and biomedical devices [1]. However, widespread commercialization of PCL has been limited due to existence of economical and complexity issue in its production. Combining PCL composites with polymers and natural fiber can cover aforesaid drawbacks. The utilization of inexpensive, renewable, accessible, and biodegradable farming residues such as rice straw, rice husks and corn stover can rationalize the cost effect [2,3]. Because of the availability of natural/bio-fibers from renewable resources, the use of bio-composites are expanding in recent years. In addition, bio-fibers are useable in both thermoplastic and thermosetting matrix composites [4,5]. Also, the natural fibers are cost effective and provide significant performance, especially when they are used in biodegradable matrix composites [4].

Among the materials science, nanoparticles and nanocomposites have received a great deal of attention from scientists, due to their small sizes and related unique properties [6,7]. Nanocomposite materials formed by metal nanoparticles that appropriately incorporated into the polymer matrix were found to be very significant due to their diversity in electrical, catalytic and optical properties. These diversities have potential applications in the fields of electronic, photonic, catalysis and bioengineering [8].

Magnetite (Fe_3_O_4_) combined with polymers/nanocomposites has unique multifunctional properties for materials, such as small sizes, biocompatibility, low toxicity, and superparamagnetism, which is applied in medical fields and magnetic recording media [9]. Therefore, magnetite plays a potential key role for providing the desired electrical and magnetic properties in the final composite.

To date, there have been no reports focused on ORS/Fe_3_O_4_/PCL-NCs preparation. Herein, we report on the preparation and characterization of ORS/Fe_3_O_4_/PCL-NCs with different percentages of ORS/Fe_3_O_4_-NCs (1.0, 5.0, 15.0, 30.0 and 60.0 wt. %) into the PCL as a polymeric matrix by solution casting method. The antibacterial activity of ORS/Fe_3_O_4_/PCL-NCs was determined against Gram-negative and Gram-positive bacteria.

## 2. Result and Discussion

### 2.1. Power X-ray Diffraction (PXRD)

**Figure 1:**
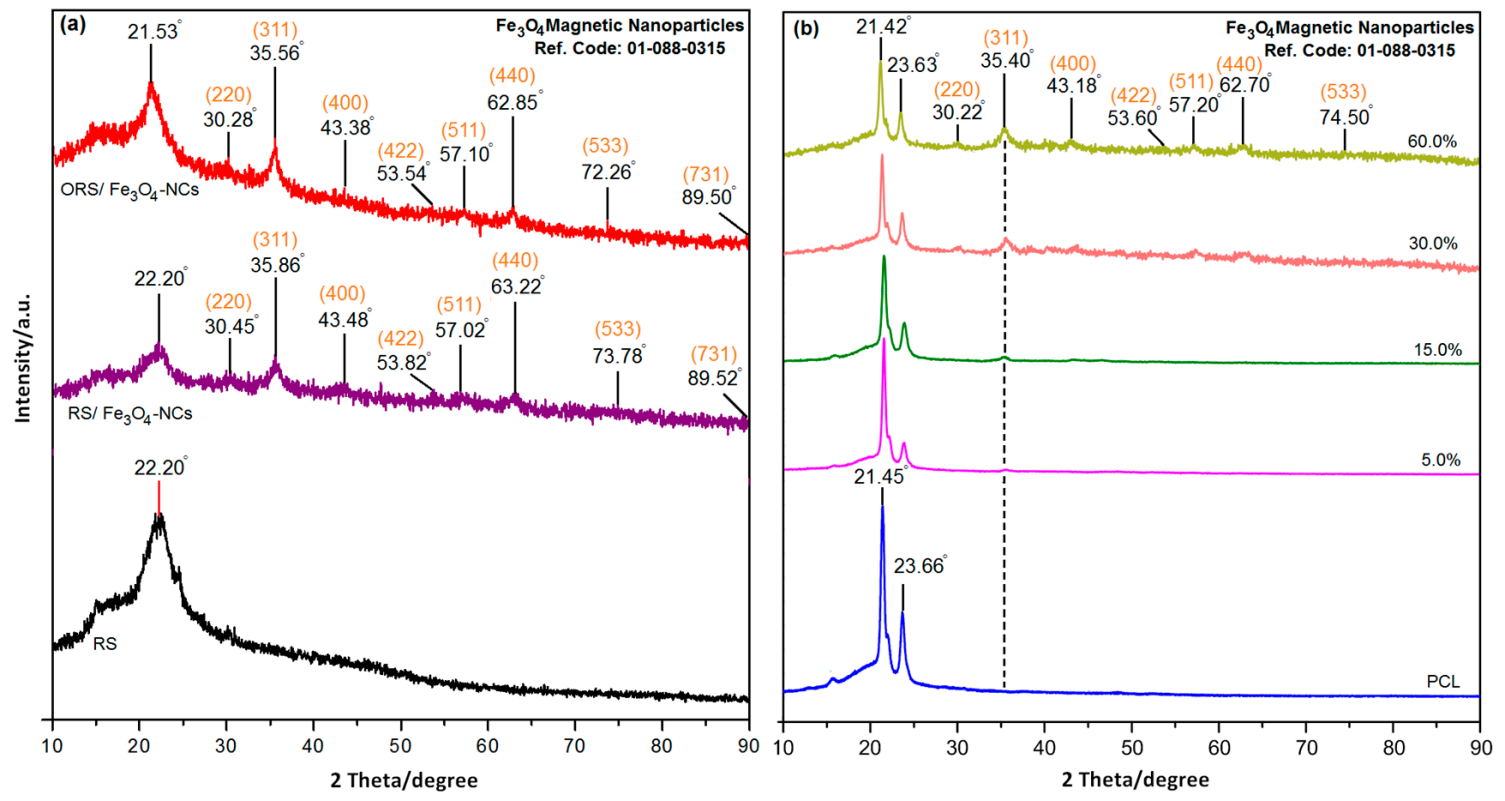
XRD pattern of RS, and RS/Fe_3_O_4_-NCs, and ORS/Fe_3_O_4_-NCs (**a**) PCL; and ORS/Fe_3_O_4_/PCL-NCs in 1.0, 5.0, 15.0, 30.0 and 60.0 wt. % (**b**).

The XRD pattern of the RS, RS/Fe_3_O_4_-NCs, ORS/Fe_3_O_4_-NCs, PCL and its nanocomposites are shown in Figure 1. A comparison of the XRD patterns of the RS and RS/Fe_3_O_4_-NCs prepared by the quick precipitation method in the small angle range of 2θ = 15° to 25° indicated the formation of nanocomposites (Figure 1a). When the percentage of Fe_3_O_4_-NPs was increased on the surface of rice straw, the intensity of these peaks decreased. The broad diffraction peak centered at 22.20° is attributed to rice straw; all the RS/Fe_3_O_4_-NCs had a similar diffraction profile, and the XRD peaks at 2θ = 30.45°, 35.86°, 43.48°, 53.82°, 57.02°, 63.22°, 73.78° and 89.52° could be attributed to the 220, 311, 400, 422, 511, 440, 533, and 731 crystallographic planes of face-centered cubic (fcc) iron crystals, respectively [10]. These peaks are consistent with the reference code Fe_3_O_4_ 01-088-0315 and reveal that the reaction product was pure Fe_3_O_4_-NPs [11].

As shown in Figure 1a, compared with the pattern of RS/Fe_3_O_4_-NCs, the diffraction peaks in ORS/Fe_3_O_4_-NCs were narrowed and the shape of peaks became sharper. This phenomenon demonstrated that alkyl ammonium might have an influence on the ORS/Fe_3_O_4_-NCs (Figure 1a). In addition, the diffraction peaks in ORS/Fe_3_O_4_-NCs at 2θ = 21.53°, 30.28°, 35.56°, 43.38°, 53.54°, 57.10°, 62.85°, 72.26° and 89.50° shifted to lower angle compared with RS/Fe_3_O_4_-NCs.

The XRD pattern of PCL (Figure 1b) showed a diffraction peak in 2θ = 21.45° and 23.66°. As can be seen from Figure 1b, with increasing amounts of ORS/Fe_3_O_4_-NCs, the height of peaks in the range of 2θ = 30° to 90° increased due to the existing nano-size particles in the nanocomposites, and the intensity of the diffraction peaks in the range of 2θ = 15° to 25° which are attributed to the PCL, was decreased when the amount of ORS/Fe_3_O_4_-NCs increased (Figure 1b). As the PCL chain was the main component of the blend, the position of crystalline peak was almost similar to that of the PCL. This shows the PCL matrix covers the RS; therefore the peaks of RS could not appear in the XRD pattern [12].

### 2.2. Morphological Studies

#### 2.2.1. Transmission Electron Spectroscopy

Transmission electron microscopy (TEM) image and their size distributions of RS/Fe_3_O_4_-NCs (not shown) showed that the mean diameters and standard deviation of Fe_3_O_4_-NPs were about 9.93 ± 2.42 nm. In addition, the uniform distribution of the Fe_3_O_4_-NPs on the surface of RS was confirmed by TEM, although particles seem to be aggregated to some extent. It can be seen that the Fe_3_O_4_-NPs exhibited spherical morphology, which agreed well with the results of XRD [13].

**Figure 2:**
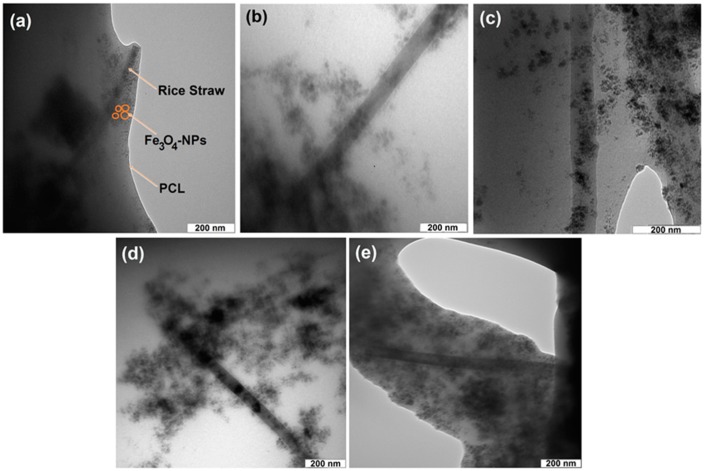
Transmission electron microscopy micrograph of ORS/Fe_3_O_4_/PCL-NCs in 1.0, 5.0, 15.0, 30.0 and 60.0 wt. % (**a**–**e**).

Figure 2 exhibited TEM images of ORS/Fe_3_O_4_/PCL-NCs with different percentages of ORS/Fe_3_O_4_-NCs. As shown in Figure 2, with increasing the ORS/Fe_3_O_4_-NCs content in the PCL from 1.0 to 60.0 wt. %, the distribution of ORS/Fe_3_O_4_-NCs was better in the polymer matrix. As shown in the images the morphological structure of ORS/Fe_3_O_4_-NCs did not change after incorporation with PCL, and the NPs exhibited spherical morphology in the PCL matrix.

#### 2.2.2. Scanning Electron Microscopy

**Figure 3:**
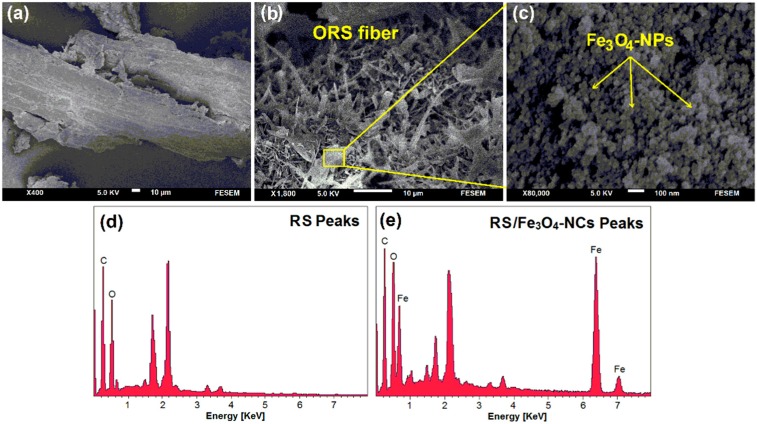
Scanning electron microscopy images of RS (**a**), ORS (**b**), RS/Fe_3_O_4_-NCs (**c**) and energy dispersive X-ray spectroscopy of RS peaks (**d**) and RS/Fe_3_O_4_-NCs peaks (**e**).

Figure 3a,c shows the surface morphology of RS and RS/Fe_3_O_4_-NCs. There were no morphological differences between the initial RS and the RS/Fe_3_O_4_-NCs. As shown in the images, RS/Fe_3_O_4_-NCs contained uniform spherical particles that indicated good dispersion of the NPs on the rice straw surface, which are in good agreement with the TEM results [11]. ORS/Fe_3_O_4_-NCs (Figure 3b) exhibited more homogenous morphology compared to the neat RS (Figure 3a) and RS/Fe_3_O_4_-NCs (Figure 3c). The homogenous dispersion of the filler and good affinity between filler-matrices led to a decreasing density of crack deflection sites and improved miscibility of polymer phases.

The chemical compositions of the RS and RS/Fe_3_O_4_-NCs were analyzed by EDX. Figure 3d shows carbon (C) and oxygen (O) peaks were observed at 0.24 and 0.4 keV in RS, respectively. After the coating of Fe_3_O_4_-NPs on the RS surface, the Fe peaks appeared in the EDX. The iron peaks (Fe) appeared in 0.68, 6.20 and 7.30 keV in all samples of RS/Fe_3_O_4_-NCs (Figure 3e) [14]. The peaks at 1.75 to 2.25 keV are related to gold which were used for sample coating. Therefore, EDX analyses provide direct evidence for adsorption of iron oxide on the surface of RS.

**Figure 4:**
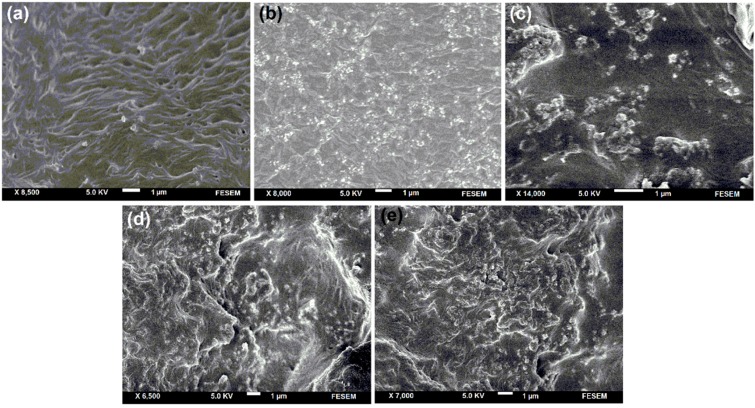
Scanning electron microscopy micrograph of ORS/Fe_3_O_4_/PCL-NCs in 1.0, 5.0, 15.0, 30.0 and 60.0 wt. % (**a**–**e**).

Figure 4a–e shows the surface morphology of ORS/Fe_3_O_4_/PCL-NCs. Excellent dispersion of ORS/Fe_3_O_4_-NCs into the PCL matrix was observed in the images. The ORS/Fe_3_O_4_-NCs did not agglomerate when modifier was used in ORS/Fe_3_O_4_/PCL-NCs. It may be due to possessing higher interfacial adhesion and smoother surface. A higher homogeneous adhesion and shiny surface were obtained in 5.0 wt. % of ORS/Fe_3_O_4_/PCL-NCs (Figure 4b). Improvment in interfacial adhesion was due to the equal hydrophobicity of the ORS/Fe_3_O_4_-NCs and PCL matrix.

### 2.3. Vibrating Sample Magnetometer

**Figure 5:**
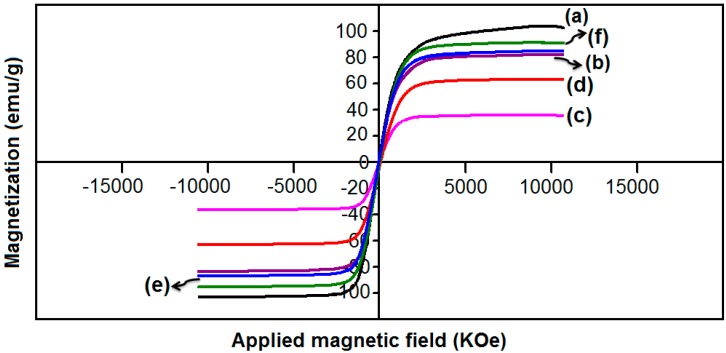
Magnetization curve of Fe_3_O_4_-NPs (a), RS/Fe_3_O_4_-NCs (b), and ORS/Fe_3_O_4_/PCL-NCs with 5.0, 15.0, 30.0 and 60.0 wt. % ORS/Fe_3_O_4_-NCs (c–f).

In order to investigate the magnetic behavior of ORS/Fe_3_O_4_/PCL-NCs, magnetization measurements with vibrating sample magnetometer (VSM) were performed. As shown in Figure 5b, it is clear, that the RS/Fe_3_O_4_-NCs exhibited superparamagnetic behavior. It also exhibited lower saturation magnetization values than the bulk Fe_3_O_4_ (~92 emu·g^−1^) (Figure 5a) [15]. The specific saturation magnetization value for RS/Fe_3_O_4_-NCs was 77.14 emu·g^−1^. As a result, the magnetic coercivity and remanence is relatively zero, which indicated superparamagnetic behavior of RS/Fe_3_O_4_-NCs. The M_s_ of the ORS/Fe_3_O_4_/PCL-NCs with 5.0 and 15.0 wt. % ORS/Fe_3_O_4_-NCs )Figure 5c,d) was 34.25 and 64.58, respectively, which is low in comparison with the bulk sample of Fe_3_O_4_-NPs. The M_s_ of the ORS/Fe_3_O_4_/PCL-NCs with 30.0 and 60.0 wt. % ORS/Fe_3_O_4_-NCs (Figure 5e,f) was 79.12 and 85.70 emu·g^−1^, respectively. The decrease in M_s_ of ORS/Fe_3_O_4_/PCL-NCs is because of the bounding of the PCL to the surface of Fe_3_O_4_-NPs which leads to pinning of some magnetic moment near the surface, so the super exchange interaction between Fe–O–Fe is weak [16].

### 2.4. FT-IR Analysis

**Figure 6:**
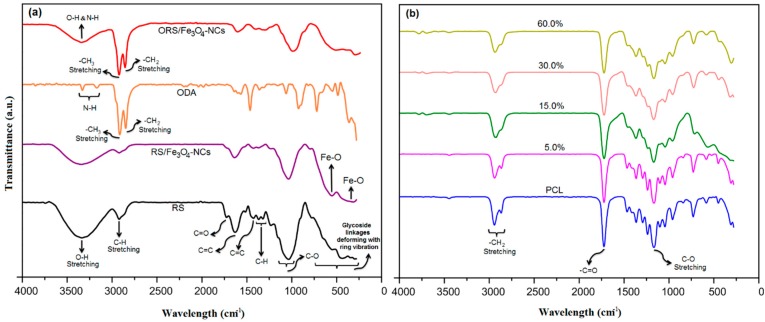
FT-IR spectra of RS, RS/Fe_3_O_4_-NCs, ODA and ORS/Fe_3_O_4_-NCs (**a**), PCL, and NCs with 5.0, 15.0, 30.0 and 60.0 wt. % ORS/Fe_3_O_4_-NCs (**b**).

The results of FT-IR spectra of RS, RS/Fe_3_O_4_-NCs, ORS/Fe_3_O_4_-NCs, PCL, and ORS/Fe_3_O_4_/PCL-NCs are summarized in Figure 6a,b. In the FT-IR spectrum of neat RS, the absorption peaks at 3377 and 2933 cm^−^^1^ were assigned to stretching vibrations of –OH groups and C–H stretching, respectively [17]. A smaller shoulder peak at 1735 cm^−1^ in the RS, is characteristic of a C=O group of the aliphatic esters in lignin or hemicelluloses. An intense band at 1646 cm^−1^ specified the olefinic C=C stretching vibration [18]. A peak at 1444 cm^−1^ is ascribed to the aromatic C=C stretch of aromatic vibration in bound lignin. The absorbance peaks at 1376–1363 cm^−1^ originated from C–H bending [19]. The region of 1200–1000 cm^−1^ represented C–O stretch and deformation bands in cellulose, lignin and residual of hemicelluloses [20]. The peaks observed in the region of 890–260 cm^−1^ is assigned to the linkages of glycoside deforming with ring vibration and OH bending [21].

The absorption bands around 295–541cm^−1^ were characteristic of Fe–O stretching and confirmed the existence of Fe_3_O_4_-NPs on the surface of rice straw via physicochemical interaction [22].

The FT-IR spectrum of ODA displayed two intense bands at 2915 and 2849 cm^−1^ that were attributed to –CH_3 _and –CH_2 _stretching. The peak at 3170 to 3331 cm^−1^ was assigned to the amino group.

In the FT-IR spectrum of ORS/Fe_3_O_4_-NCs two new intense bands at 2917 and 2851 cm^−1^ were assigned to the –CH_3_ and –CH_2_ stretching. The peak at 3347 cm^−1^ was attributed to –OH and –NH groups that overlapped with each other (Figure 6a). The FT-IR spectra demonstrated that RS/Fe_3_O_4_-NCs were modified successfully.

In Figure 6b the peaks located at 2943, 2863 and 1723 cm^−1^ were assigned to stretching vibration of –CH_2_ and vibration of –C=O bonds, respectively. The peak at 1167 cm^−1^ was related to C–O stretching [23].

There was no new peak which represented the chemical interaction between the functional groups of ORS/Fe_3_O_4_-NCs and PCL. Therefore, the interaction between ORS/Fe_3_O_4_-NCs and PCL was via a slight decrease in intensity of peaks. As shown in Figure 6b, the intensity of the peaks in ORS/Fe_3_O_4_/PCL-NCs decreased when the percentage of ORS/Fe_3_O_4_-NCs in PCL increased from 1.0 to 60.0 wt. %. It is possible that the decrease in peak intensities was due to the presence of ORS/Fe_3_O_4_-NCs in PCL matrix.

**Figure 7:**
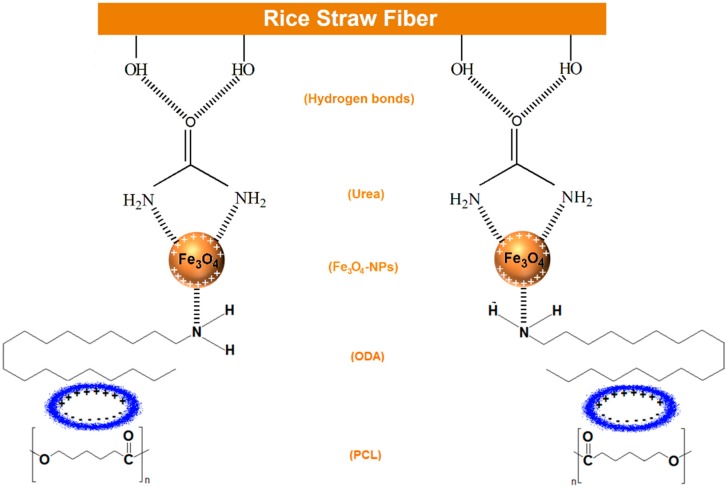
Schematic illustration of preparation of ORS/Fe_3_O_4_/PCL-NCs.

On the basis of the above results, with respect to the formation of Fe_3_O_4_-NPs, it can be seen in Figure 7 that urea was adsorbed on the surface of rice straw via hydrogen bonding between the –OH groups of rice straw and the carbonyl group of urea. Also, urea has two NH_2_ groups, which have negative dipole moments, and the surface of Fe_3_O_4_-NPs has a partial positive charge, so these two negative and positive charges can attract each other [10]. However, ODA was adsorbed on the RS/Fe_3_O_4_-NPs surface through the amine group in ODA which attracts the positive charge on the surface of Fe_3_O_4_-NPs. After that a temporary dipole moment is created between the PCL and ODA which has positive and negative charges towards ODA and PCL respectively, so these positive and negative charges can attract each other; this shows the physical interaction between RS/Fe_3_O_4_-NPs, ODA and PCL.

### 2.5. Thermal Gravimetric Analysis

**Figure 8:**
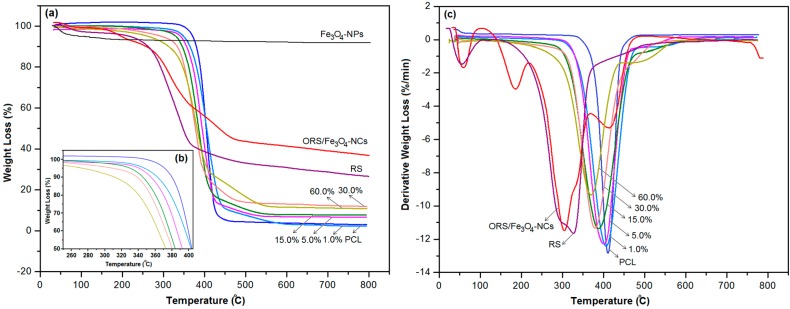
TGA (**a**,**b**) and DTG (**c**) thermograms of PCL, Fe_3_O_4_-NPs, ORS/Fe_3_O_4_-NCs and ORS/Fe_3_O_4_/PCL-NCs, with 1.0, 5.0, 15.0, 30.0 and 60.0 wt. % ORS/Fe_3_O_4_.

Thermal gravimetric analysis (TGA) of ORS/Fe_3_O_4_-NCs, Fe_3_O_4_-NPs, PCL and ORS/Fe_3_O_4_/PCL-NCs was performed to investigate the stability of NCs. TGA and differential thermal gravimetric (DTG) thermograms of the PCL, Fe_3_O_4_-NPs ORS/Fe_3_O_4_-NCs and ORS/Fe_3_O_4_/PCL-NCs are illustrated in Figure 8a,c and the degradation temperatures regarding 50.0 wt. % weight loss of PCL and its NCs are illustrated in Figure 8c. PCL has a two-step process mechanism of decompositions; in the first step (200–400 °C), random chain scission through pyrolysis of the ester groups, releases CO_2_, H_2_O and hexanoic acid. Then, in the second step (400–530 °C), ɛ-caprolactone (cyclic monomer) is constituted as a product of an unzipping depolymerization process [24].

TGA of RS shows three stages of degradation, the first stage (50–130 °C) is related to the removal of absorbed moisture; The second step of thermal degradation happens at 180–360 °C and is mainly assigned to the degradation of cellulosic materials like hemicellulose and cellulose, and the third step of the weight loss (360–480 °C) is actually related to the degradation of non-cellulosic substances in the RS. TGA of ORS/Fe_3_O_4_-NCs exhibited four stages of degradation, the first stage (50–130 °C) was related to removal of H_2_O, the second stage of degradation (131–230 °C) is attributed to the removal of ODA, and the third and fourth steps (233–500 °C) refer to the decomposition of the ORS in ORS/Fe_3_O_4_-NCs.

In the TGA curve of Fe_3_O_4_-NPs no considerable weight loss was observed for Fe_3_O_4_-NPs and about 8.0 wt. % weight loss over the temperature ranging from room temperature to 800 °C resulted from the loss of residual water in the sample (Figure 8a) [25]. Thus, there was no significant change in temperature with increasing amounts of Fe_3_O_4_-NPs.

TGA thermograms of NCs with 1.0, 5.0, 15.0, 30.0 and 60.0 wt. % ORS/Fe_3_O_4_-NCs indicated two stages of degradation (Figure 8a). Also, according to the DTG curves, all nanocomposites show two main degradation steps. The degradation of ORS occurred at the first stage, at a temperature between 260 and 440 °C, and the polymeric matrix was degraded in the second stage at a temperature between 405 and 550 °C.

It can be seen that the ORS/Fe_3_O_4_/PCL-NCs show lower onset temperature for the thermal degradation than neat PCL (Figure 8a). The lower onset temperature for the thermal degradation of NCs compared to PCL was due to the low thermal stability of ORS/Fe_3_O_4_-NCs. NCs had higher thermal stability than ORS/Fe_3_O_4_-NCs due to physical interaction between PCL and Fe_3_O_4_-NPs. On the other hand ORS/Fe_3_O_4_/PCL-NCs in higher loading percentages of ORS/Fe_3_O_4_-NCs have a lower onset temperature; this demonstrated that thermal stability decreased gradually with increasing amounts of ORS/Fe_3_O_4_-NCs. This result can be related to the weak structure of PCL caused by the expansion of PCL induced by RSF [3].

The DTG curves demonstrated that the maximum degradation rate (T_max_) of NCs is lower than the maximum degradation rate of pristine PCL. The decrease in the degradation temperature is due to the ORS/Fe_3_O_4_-NCs’s low thermal stability, which results in the heat transmission and raises the diffusion of volatile products released by the substances. This result shows the TGA and DTG are in good agreement with each other.

Table 1 shows the degradation temperature of ORS/Fe_3_O_4_-NCs, PCL, and NCs according to TGA, and DTG.

**Table 1:** Degradation temperature at 5.0%, 10.0%, 50.0% and 80.0% fiber degradation, obtained by the TGA and DTG.

Samples	T_5%_ (°C)	T_10%_ (°C)	T_50%_ (°C)	T_80%_ (°C)	T_max_ (°C)	Residue at 500 °C (%)
ORS/Fe_3_O_4_-NCs	220.43	263.56	436.32	-	408.62	44.12
PCL	370.83	380.01	404.16	418.50	409.04	5.00
1.0%	355.70	369.56	403.07	427.23	406.78	7.72
5.0%	341.31	354.48	393.25	418.16	400.02	9.09
15.0%	327.24	345.40	383.99	406.72	387.26	10.90
30.0%	318.16	331.78	380.58	408.83	379.18	14.08
60.0%	290.10	311.35	372.64	454.40	368.10	16.81

### 2.6. Iron Ion Release

**Figure 9:**
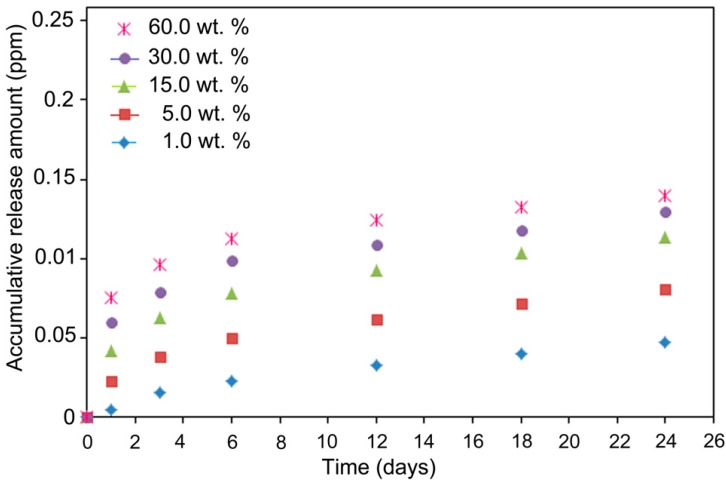
Fe^+3^and Fe^+2^ release curves of ORS/Fe_3_O_4_/PCL-NCs in PBS (pH = 7.00) with 1.0, 5.0, 15.0, 30.0 and 60.0 wt. %, respectively.

Iron ion released from the ORS/Fe_3_O_4_/PCL-NCs films were investigated in phosphate buffered saline (PBS, pH = 7.00). The released iron was detected using atomic absorption spectroscopy. Iron was identified as the cations Fe^3+^ and Fe^2+^. Therefore, the metallic iron in the polymeric matrix was converted to cationic iron during the release process through reaction with water. As the results show in Figure 9, however, while the release of Fe^3+^ and Fe^2+^ for a given iron content was relatively fast at the beginning, it became slower according to incubation time, and the releasing process can be prolonged for more than 24 days. The total amount of releasing iron depends on the iron content in the polymer films. Thus, a high quantity of initial iron content leads to a much faster release of Fe^3+^ and Fe^+2^. A steady and extended-release of iron cations can inhibit bacterial growth [26]. As a result, ORS/Fe_3_O_4_/PCL-NCs films may have antibacterial capability. The release of Fe_3_O_4_-NPs in the solution followed the Equations (1) and (2) below:

Fe_3_O_4_ → FeO + Fe_2_O_3_ (1)

(2) $$\left\{ \begin{array}{c} \;2\mathrm{FeO}\overset{{\text{H}}_{2}\text{O}}{\to }\mathrm{Fe}{\left(\text{OH}\right)}_{2}\to {\text{Fe}}_{\left(\text{aq}\right)}^{+2}+2{\text{e}}^{-}\\ {\text{Fe}}_{2}{\text{O}}_{3}\overset{3{\text{H}}_{2}\text{O}}{\to }2{\mathrm{Fe}(\text{OH})}_{3}\to {\text{Fe}}_{\left(\text{aq}\right)}^{+3}+3{\text{e}}^{-} \end{array}\right.$$

### 2.7. Mechanical Properties

#### 2.7.1. Tensile Strength

**Figure 10:**
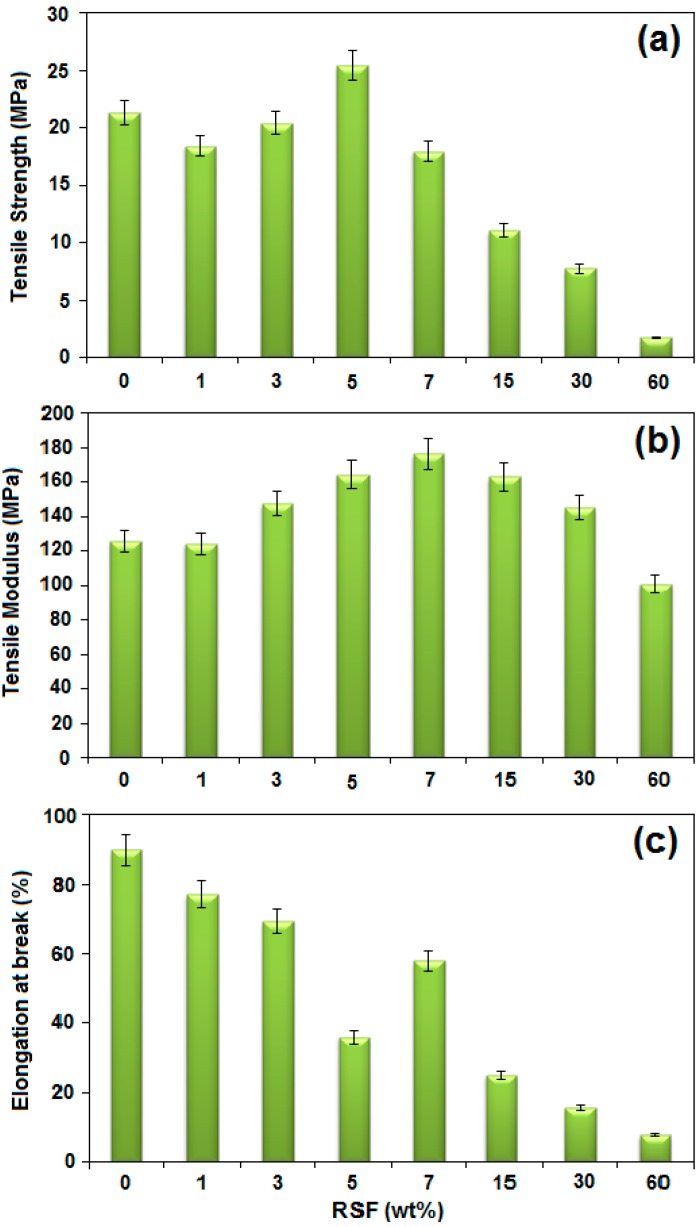
Tensile strength (**a**), tensile modulus (**b**), and Elongation at break (**c**) ORS/Fe_3_O_4_/PCL-NCs in different wt. % of ORS/Fe_3_O_4_-NCs.

The effect of ORS/Fe_3_O_4_-NCs loading on the tensile strength of ORS/Fe_3_O_4_/PCL-NCs was depicted in Figure 10a. The tensile strength of NCs increased with increasing amounts of ORS/Fe_3_O_4_-NCs up to 5.0 wt. % NCs. The highest tensile strength which was observed at 5.0 wt. % of ORS/Fe_3_O_4_-NCs loading were 25.42 MPa. This indicated good dispersion of ORS/Fe_3_O_4_-NCs in the PCL matrix. Beyond the 5.0 wt. % of ORS/Fe_3_O_4_-NCs, the tensile strength is decreased due to the saturation of filler surface by bound PCL chains and the formation of stagnant polymeric film encapsulating filler particles [8].

The decrease in tensile strength after 5.0 wt. % demonstrated that there was no improvement in the interaction between PCL and filler. Thus, only 5.0 wt. % ORS/Fe_3_O_4_-NCs was enough to enhance the tensile strength.

#### 2.7.2. Tensile Modulus

Neat PCL exhibited a tensile modulus of 125.8 MPa, and after incorporating with ORS/Fe_3_O_4_-NCs the NCs showed higher tensile modulus compared to PCL (Figure 10b). A high tensile modulus implies that the materials are rigid; therefore, more stress is needed to produce a given amount of strain, which means it resists deformation or stretch. The increase in the tensile modulus is possibly due to the restriction of the polymer chains from the interaction with the ORS/Fe_3_O_4_-NCs surface [20]. By adding filler to the PCL the tensile modulus increased to 7.0 wt. %, but above 7.0 wt. % the tensile modulus decreased. It could be assumed at higher ORS/Fe_3_O_4_-NCs content, filler agglomeration may occur which leads to the reduction of tensile modulus [27].

#### 2.7.3. Elongation at Break

As shown in the Figure 10c, the elongation of the ORS/Fe_3_O_4_/PCL-NCs decreased with increase in the amount of ORS/Fe_3_O_4_-NCs with a maximum reduction at 5.0 wt. %. This indicated that ductility of the matrix gradually decreased with increasing content of ORS/Fe_3_O_4_-NCs. The lowering of elongation was because of increasing hardness and adherence of the macromolecular chains to the surface of nanoparticles by polymer-filler interaction and, thereby, their mobility was restricted [8]. The maximum reduction in 5.0 wt. % of filler can be explained by the fact that the strong filler-matrix interaction occurred because the filler content is 5.0 wt. %. PCL matrix provided ductility whereas the ORS/Fe_3_O_4_-NCs exhibited brittle behavior with a subsequent loss of toughness in composite material.

### 2.8. Antibacterial Activity

**Figure 11:**
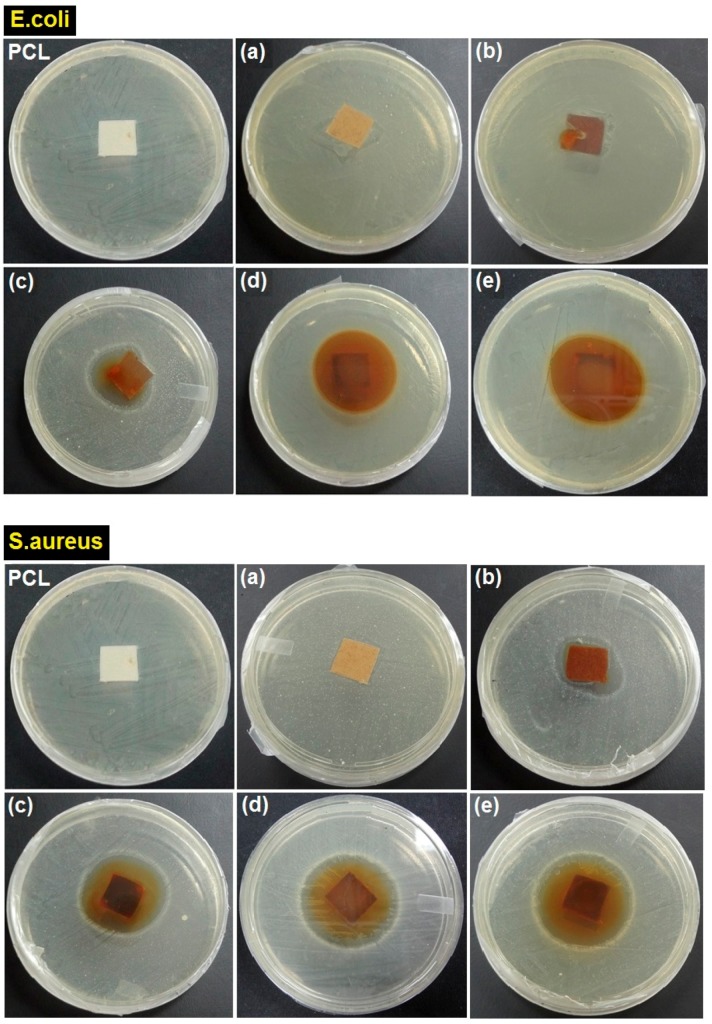
Inhibition zone of ORS/Fe_3_O_4_/PCL-NCs against Gram-negative (*E. coli*) and Gram-positive (*S. aureus*) bacteria at 1.0, 5.0, 15.0, 30.0 and 60.0 wt. % ORS/Fe_3_O_4_-NCs (**a**–**e**), respectively.

The results of antibacterial activity of ORS/Fe_3_O_4_/PCL-NCs from the agar disc diffusion method showed a remarkable inhibitory activity against *E. coli* and* S. aureus*. Table 2 shows the average diameters zone of all samples. As can be seen by the increase of ORS/Fe_3_O_4_-NCs content the antibacterial activity increased gradually. It is clearly evident from the result that the antibacterial activity of the samples was notably stronger against Gram-positive *S. aureus* than Gram-negative *E. coli.* The stronger antibacterial activity against Gram-positive bacteria is due to the structural difference in cell wall composition of Gram-positive and Gram-negative bacteria. The Gram-negative bacteria have a layer of lipopolysaccharides on the exterior, followed underneath by a layer of peptidoglycan [28]. Furthermore, this structure helps bacteria to survive in environs where exterior materials exist that can damage them. On the other hand, the cell wall in Gram-positive bacteria is principally composed of a thick layer of peptidoglycan consisting of linear polysaccharide chains cross-linked by short peptides to form a three-dimensional rigid structure. The rigidity and extended cross-linking not only endow the cell walls with fewer anchoring sites for the Fe_3_O_4_-NPs but also make them difficult to penetrate [29]. Figure 11 shows the results of the test carried out for the evaluation of antibacterial activity.

**Table 2:** Inhibition zone of ORS/Fe_3_O_4_/PCL-NCs in different percentages of ORS/Fe_3_O_4_-NPs.

Samples	Inhibition Zone (mm)	
	Gram-Positive *Staphylococccus aureus*	Gram-Negative *Escherichia coli*
PCL	-	-
1.0%	-	-
5.0%	25.3 ± 0.10	-
15.0%	31.0 ± 0.14	29.7 ± 0.23
30.0%	37.5 ± 0.21	36.2 ± 0.13
60.0%	38.4 ± 0.27	37.1 ± 0.14

## 3. Experimental Section

### 3.1. Materials

All chemicals used were of analytical reagent grade and employed without further purification. Rice straw was harvested from a local farm (Bukit Tinggi, Kedah, Malaysia). Reagents which were consumed for the synthesis of Fe_3_O_4_-NPs are as follow: urea (99%) was purchased from Chemicals Hamburg (Hamburg, Germany). FeCl_3_·6H_2_O and FeCl_2_·4H_2_O (99.89%) were supplied by Merck (Frankfurter, Germany). NaOH (extra pure) was obtained from R & M Chemicals (Chicago, IL, USA). Polycaprolactone was from (Sigma-Aldrich, St. Louis, MO, USA). Octadecylamine (ODA) (Merk, Darmstadt, Germany) was used for the modification of RS/Fe_3_O_4_-NCs. Dichloromethane (CH_2_Cl_2_) used as a solvent (QREC, Rawang, Malaysia). Phosphate buffered saline (PBS) (pH = 7.00) was supplied by JT Baker (Griesheim, Germany). All glassware used in experimental procedures were cleaned in a fresh solution of HNO_3_/HCl (3:1, *v*/*v*) and washed thoroughly with double distilled water, and dried before use.

### 3.2. Synthesis of Rice Straw/Fe_3_O_4_ Nanocomposites

For the synthesis of 20.0 wt. % RS/Fe_3_O_4_-NCs, RS (6 g) was suspended in deionized water. After that, the urea solution (20.0 mL, 2.0 M) was added to the mixture as a stabilizing agent. Iron(II) and (III) chloride salts (Fe^3+^:Fe^2+^) with a molar ratio of 2:1 were added into the modified RS mixture with vigorous agitation under inert nitrogen gas to prevent oxidation of Fe^2+^ in the mixture. Then a freshly prepared solution of NaOH (20.0 mL, 2.0 M) was then added to the mixture with a molar ratio of 1:4 to prepare iron oxide nanoparticles. The reducing agent was continually added; the reaction mixture flask was stirred for another hour. The Fe_3_O_4_-NPs were prepared at basic pH, and measured during the reaction process. The pH of the rice straw after the addition of urea was 5.71, because urea is a weak base. Then, iron chlorides and NaOH were added and the pH adjusted to 9.0. Ultimately, the suspension was centrifuged, and then washed with ethanol and deionized water (2 × 20 mL each solvent). All the precipitates were collected and dried in an oven at 60 °C. All experiments were performed at room temperature [11].

### 3.3. Modification of Rice Straw/Fe_3_O_4_-NCs

Rice straw was used as filler in this research. RS/Fe_3_O_4_-NCs (6.0 g) were dispersed into hot deionized water (150 mL, 80 °C) with continuous stirring within an hour. At the same time, ODA (4.05 g) was poured into the hot deionized water (100 mL, 80 °C) for an hour in a separate beaker. The speed of the mechanical stirrer was adjusted at 200 rpm. Then, both the above-mentioned solutions were combined. In order to flocculate the RS/Fe_3_O_4_-NCs, the reaction mixture were stirred at 80 °C for an hour. In the next step, the resulted solution was filtered carefully more than one time for each gram of RS/Fe_3_O_4_-NCs in content. After each filtering cycle, the precipitate was collected, washed with hot deionized water and stirred in that water at the speed of 200 rpm.

### 3.4. Preparation of Modified RICE Straw/Fe_3_O_4_/Polycaprolactone Nanocomposites

For the synthesis of ORS/Fe_3_O_4_/PCL -NCs, different ratios of ORS/Fe_3_O_4_-NCs (1.0, 5.0, 15.0, 30.0 and 60.0 wt. %) were suspended in certain amounts of dichloromethane with stirring for half an hour, and then 5.0 g PCL was dissolved in 50.0 mL dichloromethane, and the ORS/Fe_3_O_4_-NCs suspension was added slowly to a PCL solution with vigorous stirring. After addition of the ORS/Fe_3_O_4_-NCs, the suspension was stirred for a further hour to allow the ORS/Fe_3_O_4_-NCs to be well dispersed in the PCL matrix. The suspensions were finally poured in petri dishes and kept for 2 days until completely dry. Finally the solidified films, with a thickness of about 0.5 mm were obtained. In this step, all experiments were carried out at room temperature.

### 3.5. Iron Ions Release

The films of ORS/Fe_3_O_4_/PCL-NCs were prepared in certain pieces (1.0 cm × 1.0 cm) for identifying the released iron ions test. In vitro release test of iron was carried out in 40.0 mL of PBS. The samples were incubated at 37 °C under water shaker at 70.0 rev·min^−1^. A small amount of sample was withdrawn from the flask and the iron concentration was measured by atomic absorption spectroscopy. Sample withdrawal was operated for 24 days.

### 3.6. Evaluation of Antibacterial Activity

The disc diffusion method was used to screen the antibacterial activity. *In vitro* antibacterial activity was screened by using nutrient agar (NA). The inhibition zone in millimeters (mm) was determined based on the recommended standards of the National Committee for Clinical Laboratory Standards. The antibacterial activity of ORS/Fe_3_O_4_/PCL-NCs films was scrutinized against pathogenic Gram-negative bacteria, *Escherichia coli* and Gram-positive bacteria, *Staphylococcus aureus* at different percentages of ORS/Fe_3_O_4_-NCs in the polymeric matrix. Square samples (1.5 cm × 1.5 cm) of PCL and ORS/Fe_3_O_4_/PCL-NCs films containing different percentages of ORS/Fe_3_O_4_-NCs were sterilized by immersion in ethanol for 10 min and placed on the surface of NA which was seeded per 1.0 mL of microorganism culture. The plates were inoculated at 37 °C for 24 h. The diameters of the zone of inhibition around the film specimen were used to determine the antibacterial activity of each film sample, and the average of 3 replicates was recorded.

### 3.7. Characterization

Transmission electron microscopy (TEM) was applied to measure the morphology and size of the obtained samples. A drop of diluted sample in deionized water and dichloromethane was dripped on a covered copper grid. TEM observations were performed using a Hitachi H-7100 electron microscope. Electron field emission scanning electron microscopy (FESEM) was applied to observe the morphology of the RS, ORS/Fe_3_O_4_-NCs, PCL and ORS/Fe_3_O_4_/PCL-NCs. FESEM was performed utilizing JEOL, JSM-7600F instrument. The powder X-ray diffraction (XRD) with Cu Kα radiation was used to measure the crystallinity of samples. The thermal behavior of the samples was measured by Thermo gravimetric analysis (TGA) and differential thermal gravimetric (DTG) instruments. Fourier transform infrared (FT-IR) in the range of 400–4000 cm^−1^ was used in order to study the structure of the RS, ORS/Fe_3_O_4_-NCs, ODA, PCL and ORS/Fe_3_O_4_/PCL-NCs. FT-IR Spectra were recorded using Series 100 PerkinElmer FT-IR 1650 spectrophotometer. Tensile strength, Young’s modulus and elongation at break were measured using the Instron Universal Testing Machine model INSTRON 4302 at constant cross-head speed of 5 mm/min and 1 kN load. Four samples were employed for the tensile test and the average values were calculated from five runs for each sample. The released Fe^2+^ and Fe^3+^ concentrations in PBS solution were determined using atomic absorption spectrometer (Thermo Scientific, S. Series).

## 4. Conclusions

ORS/Fe_3_O_4_/PCL-NCs with various percentage loadings of ORS/Fe_3_O_4_-NCs were successfully prepared through a solution casting of PCL and ORS/Fe_3_O_4_-NCs. The properties of ORS/Fe_3_O_4_/PCL-NCs were investigated. The XRD analysis shows ORS/Fe_3_O_4_-NCs were modified successfully with ODA and resulted in the shifting in small 2θ angle of ORS/Fe_3_O_4_/PCL-NCs implying the formation of NCs. The SEM and TEM images show a good dispersion of ORS/Fe_3_O_4_-NCs in the polymer matrix. FT-IR results show no chemical interaction between PCL and ORS/Fe_3_O_4_-NCs, and the interaction could be a physical interaction as there is no new band or any significant shift compared to the PCL spectrum. TGA thermogram shows thermal stability decreased with increasing ORS/Fe_3_O_4_-NCs content. The optimum ORS/Fe_3_O_4_-NCs loading with enhanced mechanical properties of PCL was 5.0 wt. % ORS/Fe_3_O_4_-NCs. The antibacterial properties of ORS/Fe_3_O_4_/PCL-NCs show that activity against Gram-positive bacteria is higher than that against Gram-negative bacteria.
